# Efficient generation of functional pancreatic β cells from dental-derived stem cells via laminin-induced differentiation

**DOI:** 10.1186/s43141-022-00369-6

**Published:** 2022-06-08

**Authors:** Riham M. Aly, Hadeer A. Aglan, Ghada Nour Eldeen, Nadia S. Mahmoud, Eman H. Aboul-Ezz, Hanaa H. Ahmed

**Affiliations:** 1 Basic Dental Science Department, Oral Medicine & Dentistry Research Institute, National Research Centre, Dokki, Giza, Egypt; 2 Stem Cell Laboratory, Center of Excellence for Advanced Sciences, National Research Centre, 33 El Buhouth St., Dokki, 12622 Giza, Egypt; 3 Hormones Department, Medicine Research and Clinical Studies Institute, National Research Centre, Dokki, Giza, Egypt; 4 Molecular Genetics & Enzymology Department, Human Genetic & Genome Research Institute, National Research Centre, Dokki, Giza, Egypt

**Keywords:** Dental stem cells, Insulin-producing cells, Diabetes mellitus, Laminin, Differentiation

## Abstract

**Background:**

This study was designed to generate functional insulin-producing cells (IPCs) from dental-derived mesenchymal stem cells (MSCs) and further explore their therapeutic potential against diabetes mellitus in vivo. MSCs were isolated from human dental pulp and periodontal ligament and were induced to differentiate into insulin-producing cells (IPCs) using laminin-based differentiation protocol for 14 days. Confirmation of IPCs was performed through real-time PCR analysis and insulin release assay. Then, the generated IPCs were labeled with PKH26 dye prior to transplantation in experimental animals. Twenty-eight days later, blood glucose, serum insulin (INS), c-peptide (CP), and visfatin (VF) levels and pancreatic glucagon (GC) level were estimated. Pancreatic forkhead box protein A2 (Foxa2) and SRY-box transcription factor 17 (Sox17), insulin-like growth factor I (IGF-1), and fibroblast growth factor10 (FGF 10) gene expression levels were analyzed.

**Results:**

Dental stem cells were successfully differentiated into IPCs that demonstrated increased expression of pancreatic endocrine genes. IPCs released insulin after being subjected to high levels of glucose. In vivo findings uncovered that the implanted IPCs triggered significant decrease in blood glucose, serum VF, and pancreatic GC levels with significant increase in serum INS and CP levels. Furthermore, the implanted IPCs provoked significant upregulation in the expression level of pancreatic genes. Histopathological description of the pancreas tissues revealed that transplantation of IPCs ameliorated the destabilization of pancreas tissue architecture.

**Conclusion:**

This study demonstrates the significant role of the implantation of IPCs generated from dental-derived stem cells in treatment of diabetes mellitus.

## Background

Diabetes mellitus (DM) is a debilitating disease that affects millions of people and results in serious complications that could lead to systemic impairment and even mortality. Currently, available therapies, vary from insulin-based treatments to beta cell replacement altogether either via the pancreas or pancreatic islet transplantation. However, such interventions are limited by the availability of the organs. Stem cell-based therapies offer an alternative treatment option for diabetes through sequential differentiation of stem cells into beta cells that are able to react to blood glucose and consequently synthesize and secrete insulin [6].

β cells differentiated from mesenchymal stem cells are considered the most auspicious source of cells for diabetic cell based therapies [34, 55]. Dental-derived stem cells in particular are undoubtedly considered an attractive source of MSCs [1]. They are easy to obtain through non-invasive procedures where the donors suffer no discomfort. Furthermore, there is no ethical controversy involved in acquiring the teeth, since dental stem cells are usually isolated from the teeth that are originally indicated for extraction whether due to impaction or for orthodontic treatment purposes [27, 46, 59].

Several differentiation protocols have been devised, which lead to differentiation of MSCs into insulin-producing cells (IPCs). These differentiation protocols work either through genetic manipulation of the cells or through the epigenetic effects elicited by the factors comprising the differentiation media. However, it should be noted that differentiation of stem cells into β cells in vitro is not a single step procedure, but rather a sequential series of steps that mimics pancreatic development [40].

There is compelling evidence that indigenous extracellular matrix (ECM) promotes stem cell survival and to regulate stem cell differentiation through maintaining its inherent structural, biochemical, and biomechanical properties [28, 38, 50]. Laminin specifically, which is a major constituent of the basement membrane, plays a role in supporting cell proliferation and differentiation [22]. During organ development, laminins interplay with several other ECM molecules and connect cells through cell surface receptors, which results in modulating cellular phenotypes and differentiation [24, 51, 53]. Recent studies reported that, laminin-411 promoted islet function in the developing pancreas. Moreover, it proved to be found crucial for beta cell proliferation and insulin maintenance [43]. Laminins also induce expression of islet-specific factors like pancreatic and duodenal homeobox 1 (PDX1), insulin 1, insulin 2, glucagon, somatostatin, and GLUT-2. They also activate protein kinase B and extracellular signal-regulated kinase, which are major factors in cell metabolism that are capable of inducing differentiation of beta cells [60].

In this study, we investigated the effect of Laminin 411 on the differentiation of human dental pulp stem cells (hDPSCs) and human periodontal ligament stem cells (hPDLSCs) into IPCs through applying a previously published protocol intended for human umbilical cord stem cells. Moreover, the therapeutic effect of the acquired IPCs was also validated on DM animal model.

## Methods

### Isolation and propagation of mesenchymal stem cells

#### Isolation and preparation of human dental pulp stem cells (hDPSCs)

Human third molar teeth indicated for extraction were collected from patients aged (16-24) after obtaining an informed consent. Extraction of teeth (*n*=3) was carried out at the National Research Centre’s clinics under the approval of the Centre's ethical committee. The extracted teeth were kept in Dulbecco modified Eagle’s media (Lonza) to which penicillin/streptomycin (Lonza), and 10% of FBS (Life Science Group) were added. The teeth were split open, and the pulp was gently removed using sterile dental probe. The pulp tissue was then carefully dissected into small pieces using sterile scissors. 0.2% collagenase II (Serva Electrophores, Germany) solution was then added to the dissected pulp tissue and shaken in a water bath shaker at 37°C for 30 min. The isolated dental pulp cells were cultured in fresh DMEM media supplemented with 15% FBS and incubated at 37°C and 5% CO_2_. Cells were selected on the basis of their ability to adhere to the tissue culture plastic; non adherent cells were removed during medium replacement. The medium was changed twice per week thereafter. Dental pulp cells were subsequently be detached using 0.25% trypsin/1-mM ethylene diaminetetraacetic acid (EDTA) re-plated at 5 × 10^3^ cells/cm^2^ and cultured then passaged at 70 to 80% confluency.

#### Isolation of human periodontal ligament stem cells (hPDLSCs) cells

Healthy human impacted third molars (*n*=3) (free of inflammation) were placed in 50-ml sterile polypropylene tube containing alpha modified Eagle’s medium (alpha-MEM) supplemented with antibiotics (100 U/ml penicillin and 100 mg/ml streptomycin) immediately after extraction. The periodontal ligament tissue was removed from the surface of the root by sterile lancet, rinsed five times by PBS supplemented with penicillin and streptomycin. Periodontal ligament pieces were minced into tiny pieces, then digested in a solution of 2 mg/ml collagenase type II (Serva Electrophores, Germany) for 30 min at 37°C in a water bath. Single-cell suspensions were seeded into a T-25 flask with D-MEM (Lonza), supplemented with 15% fetal bovine serum (Lonza), 20,000 U/ml penicillin and 20,000 ng/ml streptomycin and then incubated in of 5% CO_2_ at 37°C. The flasks were periodically monitored by the phase contrast-inverted microscopy and the culture medium was changed 3-times per week, and the cells were sub-cultured when about 80% confluency was reached using 0.25% trypsin EDTA.

### hDPSC and hPDLSC characterization

#### Morphological follow up by inverted microscope examination

Morphological changes were observed via an inverted light microscope (Leica, 6000B-4) using Suite V3 (Leica). Cells were regularly monitored and images were captured.

#### Flow cytometry analysis

To ensure that the cells in cultures were MSCs that maintained their phenotype after propagation in culture [12], the isolated cells were characterized by flow cytometry analysis for MSC-specific markers (CD73, CD105) in addition to hematopoietic markers (CD34 and CD45). The PE conjugated-CD 73 and FITC conjugated-CD 105 antibodies were procured from R&D Systems (UK). While the PE conjugated-CD 34 as well as FITC-conjugated CD 45 antibodies were purchased from Beckman Coulter Co. (USA). The cells were incubated with the antibody against each of the surface markers for 20 min at 25°C followed by flow cytometry analysis using Beckman Coulter Elite XL, USA, instrument.

#### Tri-lineage mesodermal differentiation

Both hDPSCs and hPDLSCs were differentiated into adipocytes upon culturing them with StemPro® adipogenesis differentiation medium provided by Gibco, Thermo Fisher Scientific Inc. (USA), for 14 days. Thereafter, adipogenic cultures were processed for Oil Red O staining (Sigma, USA) to detect the presence of highly refractive intracellular lipid vacuoles containing accumulated lipid droplets. Chondrocyte differentiation was carried out in turn by culturing the isolated MSCs with StemPro® chondrogenesis differentiation medium provided by Gibco, Thermo Fisher Scientific Inc. (USA). After 14 days under differentiating conditions, the cells were fixed with 4% formaldehyde solution for 30 min. After fixation, the wells were rinsed with PBS and stained with 1% Alcian Blue (Sigma, USA) solution prepared in 0.1 N HCL for 30 min. The wells were visualized under light microscope. Blue staining indicated synthesis of proteoglycans by chondrocytes. As for the osteogenic differentiation potential, the isolated MSCs were induced into the osteogenic pathway through culturing in StemPro® osteogenesis differentiation medium provided by Gibco, Thermo Fisher Scientific Inc. (USA). After 21 days, the cells were fixed with 4% formaldehyde solution for 30 min. The wells were then rinsed with PBS, and the cells were stained with 2% Alizarin Red S (Sigma, USA) solution (pH 4.2) for 2–3 min. The wells were visualized under light microscope for presence of calcium-rich mineralized nodules.

### Differentiation of hDPSCs & hPDLSCs into insulin-producing cells (IPCs)

**Fig. 1:**
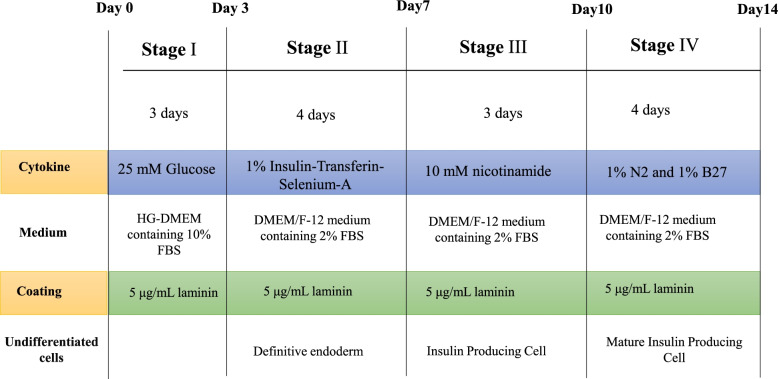
Schematic diagram outlining the differentiation protocol for dental derived stem cells (hDPSCs & hPDLSCs) into insulin-producing cells (IPC). Durations and required factors for each differentiation step are outlined

After the third passage, both hDPSCs and hPDLSCs were induced to differentiate into IPCs (Fig. 1). The protocol for differentiation was implemented as described by Qu et al. [43]. Both hDPSCs and hPDLSCs were suspended in complete culture media, and aliquots of 2.5 × 10^5^ cells were placed in 6-well plates pre-coated with 5 μg/mL laminin (Gibco) overnight. The medium was replaced with HG-DMEM containing 10% FBS for 3 d (stage I). At stage II, the media were refreshed with DMEM/F-12 (Lonza) medium containing 2% FBS and 1% Insulin-Transferin-Selenium-A (ITS-A, Gibco) for another 4 days. At stage III, 10-mM nicotinamide was added into the media described in stage II, and the culture lasted for 3 days. At stage IV, the medium was changed with the same supplements as at stage III, but 1% N2 and 1% B27 supplements (STEMCELL Technologies Inc.) were added and incubated for 4 days.

### Determination of the efficiency of differentiation of hDPSCs and hPDLSCs into insulin-producing cells (IPCs)

#### Quantitative real-time PCR analysis of IPCs

The total RNA was isolated from the IPCs generated from hDPSCs and hPDLSCs using Trizol (Invitrogen) according to the manufacturer’s instructions. The first-strand cDNA was synthesized with 50 ng of the total RNA by random hexamer priming using high-capacity cDNA synthesis kit (Qiagen) at 42°C for 60 min and at 70°C for 5 min. The quantitative real-time PCR was performed in triplicates using SYBR-green kit (Qiagen) according to the manufacturer’s instructions and using the Roche LC480 in a total reaction volume of 20 μl. Results were normalized to GAPDH (internal control) to correct RNA input in reactions. All reactions were performed by annealing at 58–60°C for 40 cycles, and the melt curve analysis was achieved at the end of each reaction. The primer sequences of the target genes are listed in (Table 1).

**Table 1:** List of human gene-specific primers in RT-PCR

Gene	Forward	Reverse
**Foxa-2**	**GGAGCGGTGAAGATGGAAGG**	**CGGCGTTCATGTTGCTCAC**
**Sox-17**	**CAAGATGCTGGGCAAGTC**	**TGGTCCTGCATGTGCTG**
**PDX-1**	**ATGGATGAAGTCTACCAAAGC**	**CGTGAGATGTACTTGTTGAATAG**
**Ngn-3**	**AGAGAGCGTGACAGAGGC**	**GCGTCATCCTTTCTACCG**
**Insulin**	**GCTTCTTCTACACACCCAAG**	**GGTAGAGGGAGCAGATGC**
**Glucagon**	**ACCAGAAGACAGCAGAAATG**	**GAATGTGCCCTGTGAATG**
**GAPDH**	**GCACCGTCAAGGCTGAGAAC**	**TGGTGAAGACGCCAGTGGA**

#### Insulin release assay

The generated IPCs from both hDPSCs and hPDLSCs were gently washed twice with phosphate buffer saline (PBS, Biowest, France). Then, the cells were pre-incubated in culture media containing glucose (5.5 mM) at 37°C for 24 h [52], and supernatants were then collected for insulin quantification via ELISA technique using the kit procured from Epitope Diagnostics, Inc. (USA), according to the manufacturer’s instructions.

### Labeling of the generated IPCs with PKH26 dye

The generated IPCs were harvested and labeled with PKH26 (Sigma-Aldrich, USA) fluorescent linker dye according to the manufacturer’s instructions prior to transplantation in experimental animals. The pancreatic tissues were examined using fluorescence microscope to detect and trace the cells. PKH26-derived fluorescence was detected by inverted fluorescent microscope (Olympus, CKX41, Japan).

### Induction of DM and treatment of diabetic rats with IPCs

#### Animals

In total, 40 adult male rats (*Sprague Dawley*) weighing 150–170 g were supplied from a breading stock maintained in the Animal House of the  National Research Centre, Giza, Egypt. The animal house was ventilated with a 12-h light/dark cycle at the ambient temperature of 25–30°C throughout the experimental period with free access to tap water and a standard rodent chow (Meladco Co.). Rats were allowed to adapt to their environment for at least 2 weeks before the initiation of the experiment. Housing and management of animals and experimental protocol followed the guidelines of animal experiments and was approved by the Ethical Committee of the  Medical Research of the National Research Centre, Egypt (approval no. 19 109).

#### Study protocol

Ten rats served as the negative control group and received no drug (Group 1), while other rats were included in the induction of diabetes mellitus. Briefly, diabetes was induced via a single subcutaneous injection of streptozotocin (STZ, Sigma, USA) (50 mg/kg) following an overnight fast. Streptozotocin was dissolved in a 50-mM sodium citrate solution (pH 4.5) containing 150 mM NaCl. Fasting blood sugar was estimated after 3 days to confirm the development of diabetes mellitus using the kit supplied from Chemelex S.A. (Spain) [19]. Animals showing blood glucose levels above 250 mg/dL were included in the study.

Thereafter, diabetic animals were randomly divided into 3 experimental groups and were left without treatment (Group 2, *n* =10, DM untreated), transfused via tail vein with 5 × 10^6^ cells/rat of IPCs generated from human DPSC differentiation (Group 3, *n* = 10, DM + hDPSCs), and finally transfused via tail vein with 5 × 10^6^ cells/rat of IPCs generated from human PDLSCs differentiation (Group 4, *n* = 10, DM + hPDLSCs).

Twenty-eight days following the infusion of IPCs, the diets were withheld from the experimental rats for 12 h and then blood samples were collected, under diethyl ether anesthesia, from the retro-orbital venous plexus in clean centrifuge tubes and allowed to coagulate at room temperature. Serum samples were separated by centrifugation at 1800×*g* for 15 min at 4°C using cooling centrifuge for biochemical measurements and retained at −20°C till analysis. After the collection of blood samples, the rats were euthanized by cervical dislocation and a middle abdominal incision was performed and the pancreas of each rat were quickly dissected out and washed in ice cold saline. Some of pancreas tissues were immediately weighed and homogenized in ice-cold medium containing 50-mM Tris-HCl (pH 7.4) to give 10% homogenate (W/V) [19]. While others were immediately frozen in liquid nitrogen and kept at −80°C preceding the molecular genetics investigation. Lastly, the rest of the pancreas was placed in neutral-buffered formalin (10%) to be used for histopathological investigations.

### Evaluation of IPC therapeutic efficacy of the generated IPCs

#### Biochemical analysis

Serum glucose level was estimated using the kit supplied from Chemelex S.A. (Spain). While serum insulin (INS), c-peptide (CP), and visfatin (VF) levels were determined by ELISA using the specific kits for rat supplied from SinGeneClon Biotech Co., Ltd, (China) according to the manufacturer’s manuals. Pancreatic glucagon (GC) level was determined by ELISA using the rat specific kit procured from SinGeneClon Biotech Co., Ltd, (China) following the manufacturer’s instructions.

#### Molecular genetic analysis

The total RNA was isolated from the pancreatic tissue of rats of different experimental groups using Trizol reagent (Invitrogen, USA. Cat #15596-018) in combination with RNeasy mini kit for total RNA purification from animal cells (Cat#74104, Qiagen, Germany) according to the manufacturer’s instruction. The RNA integrity was evaluated using Nano Drop 2000 (Thermo Fisher Scientific, Rockford, IL, USA) using 260/280 nm ratio. Then, the cDNA synthesis was performed using Revert Aid first-strand cDNA synthesis kit (Cat# K1621, Thermo Fisher Scientific, Inc., Lithuania) according to the manufacturer’s instruction. Gene expression analysis of forkhead box protein A2 (Foxa2) and SRY-box transcription factor 17 (Sox17), insulin-like growth factor I (IGF-1), and fibroblast growth factor (FGF) 10 was carried out in rat pancreatic tissues using DNA-Technology Real-Time PCR device (DT lite 4, Russia). The reaction mixture (25 μl volume) included 12.5 μl of QuantiTect SYBR Green master mix (Cat# 204141, Qiagen, Germany), 0.75 μl of forward and reverse primer of target gene (Invitrogen, USA), cDNA template (100 ng), and RNase free water. GAPDH was used as a housekeeping gene. Relative mRNA expression versus control value was assessed using the 2^-ΔΔCt^ comparative method after normalization with GAPDH gene. The PCR cycling was set as follows: initial denaturation step at 94°C for 15 min, followed by 40 cycles of denaturation at 94°C for 15 s, annealing at 60°C for 30 s, and extension at 72°C for 30 s for 5 min. The primer sequences of each target gene are delineated in Table 2. The data were presented as the fold change in gene expression level in the diabetic group relative to the control group. While the data were represented as the fold change in gene expression as compared to diabetic group in case of treated groups.

**Table 2:** List of gene-specific primers in RT-PCR

Gene name	Forward	Reverse
**Foxa2**	TGAAGCCCGAGCACCATTAC	CCAGGGTAGTGCATGACCTGTT
**Sox 17**	GGCGCCAGCCGGGACCTC	GGCCGCCCTCGGGACCAA
**IGF-1**	GCTTTTACTTCAACAAGCCCACA	TCAGCGGAGCACAGTACATC
**FGF 10**	TTGCTCTTCTTGGTGTCTTCC	ACCTTGCCGTTCTTTTCAATC
**GAPDH**	CACCCTGTTGCTGTAGCCATATTC	GACATCAAGAAGGTGGTGAAGCAG

### Histopathological procedure

The pancreas tissues were fixed in 10% neutral-buffered formalin for 24 h, washed with tap water, and prepared and stained for light microscopy. For dehydration, serial dilutions of alcohol were applied, and thereafter, the specimens were cleared in xylene and embedded in paraffin wax in hot air oven at 56°C for 6 h. The paraffin wax tissue blocks were sectioned by using microtome at 5–6 micron thickness. Then, sections were collected on glass slides and de-paraffinized. They were stained for routine histological examination using hematoxylin and eosin stain [11].

### Statistical analysis

The experimental results were represented as arithmetic means with their standard deviation (SD). Data were statistically analyzed by one-way analysis of variance (ANOVA) test using the Statistical Package for the Social Sciences (SPSS) 14 followed by determining the least significant difference (LSD) to evaluate the significance between groups. *P* < 0.05 was considered significant.

## Results

### Morphological appearance of isolated mesenchymal stem cells hDPSCs & hPDLSCs

**Fig. 2:**
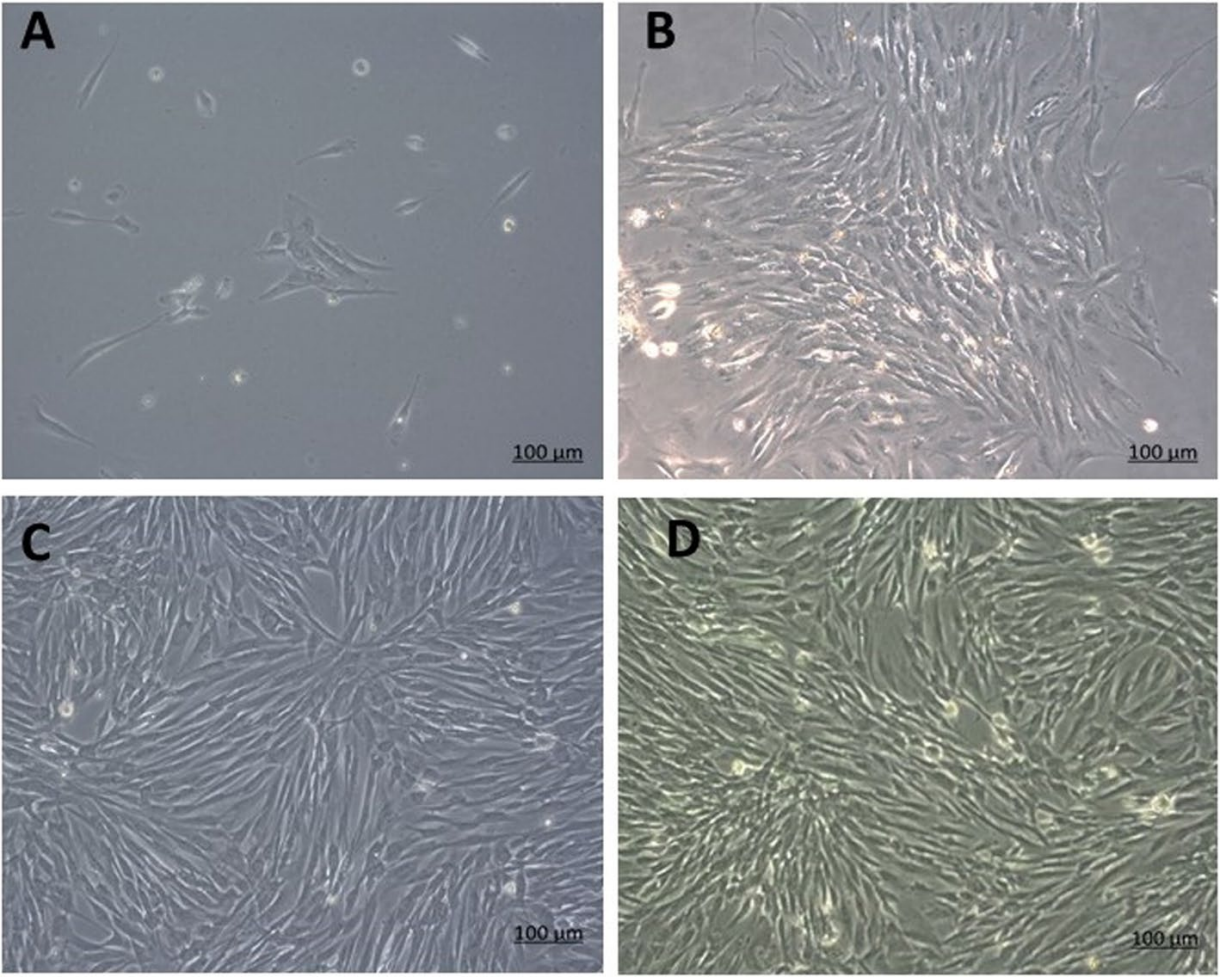
Photomicrograph illustrating the morphological changes of hDPSCs & hPDLSCs following isolation. In both cultures of hDPSCs and hPDLSCs after approximately 3 days of culture, scattered single cells started to attach to the culture plate assuming a spindle-shaped appearance (**A**). Gradually, these spindle-shaped cells started to increase in number, and colonies were evident by day 5 of culture (**B**). These colonies increased in size and reached 80% confluence by the 10th day of culture in hDPSCs (**C**) and by day 15 in hPDLSCs (**D**) (scale bar 100μm)

The photomicrographs presented in Fig. 2 illustrate the morphological appearance of cells after isolation. Images indicated that MSCs derived from human dental pulp and periodontal ligament exhibited spindle-shaped, fibroblastic-like morphology which is characteristic to MSCs.

### Mesenchymal stem cell surface markers

**Fig. 3:**
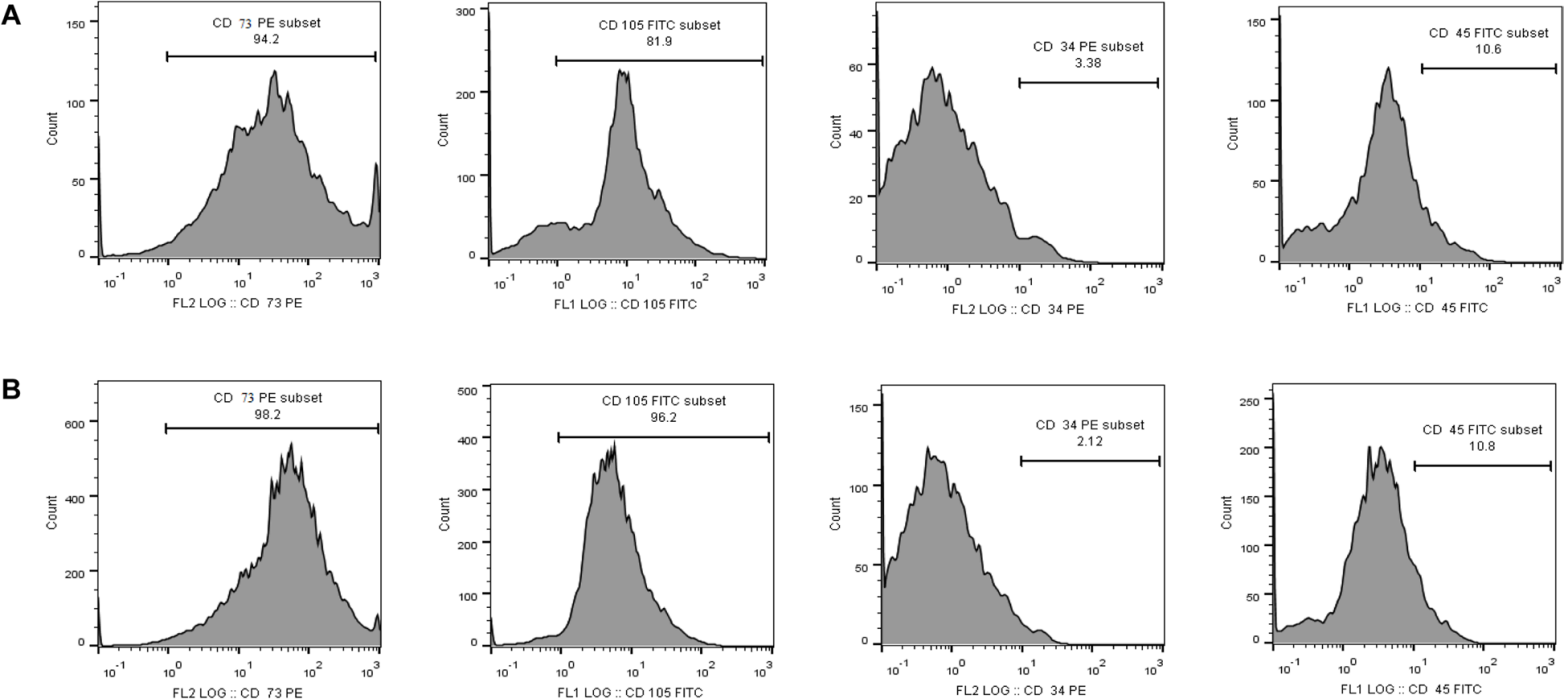
Flow cytometry analysis of cell surface markers. Mesenchymal stem cell surface markers; CD 73 and CD 105 were positively expressed in both hDPSCs and hPDLSCs whereas hematopoietic markers CD 34 and CD 45 were negatively expressed in both cell types

Both human dental pulp and human periodontal ligament-derived MSCs characterized by flow cytometry illustrated positive expression of mesenchymal stem cell markers CD 73 and CD 105 and negative expression of non-mesenchymal stem cell markers CD 34 and CD 45 (Fig. 3).

### Tri-lineage mesodermal differentiation

**Fig. 4:**
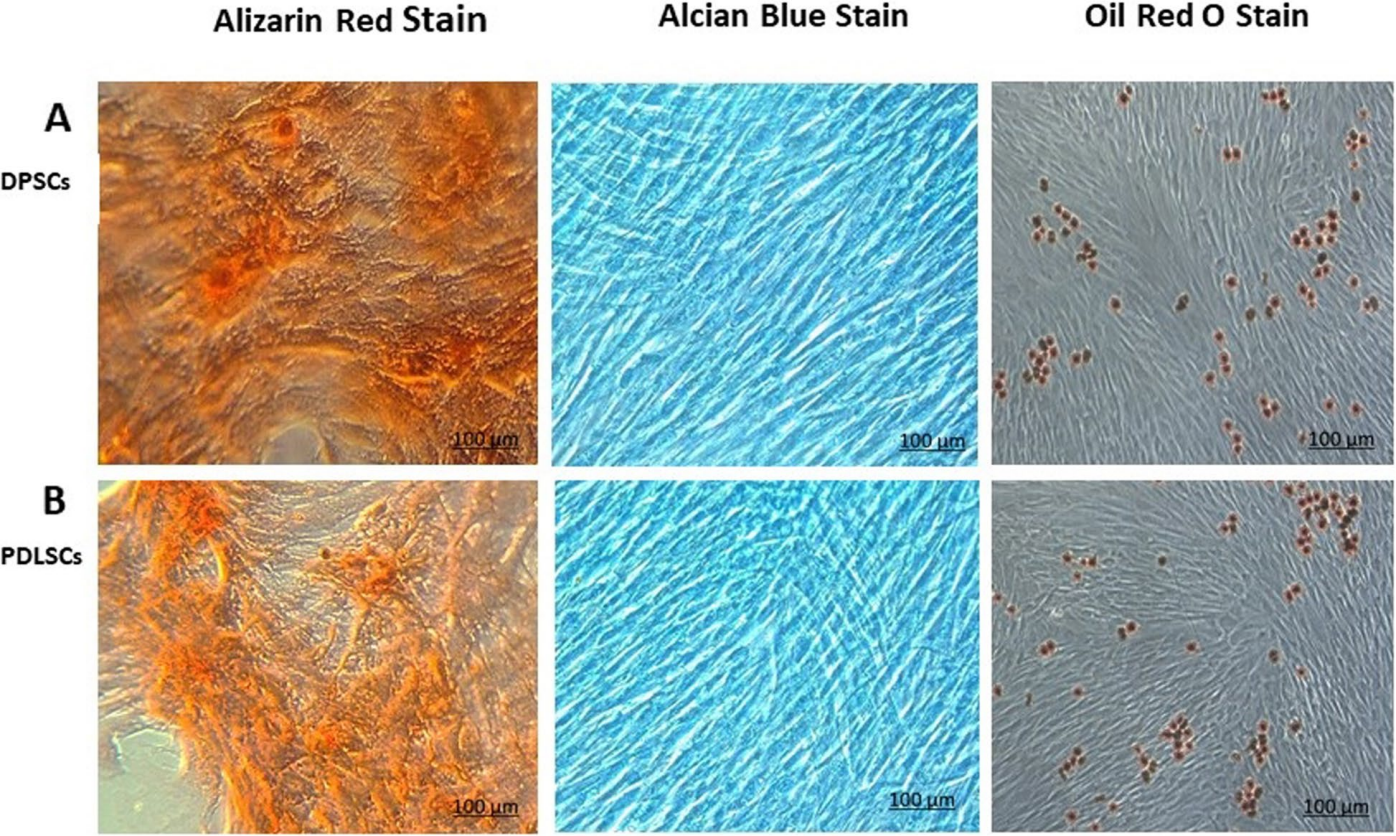
Multi-lineage differentiation of hDPSCs (**A**) and hPDLSCs (**B**). In order to confirm the stem cell identity of the isolated cells from dental pulp and periodontal ligament, multi-lineage differentiation potential of those cells into adipogenic, chondrogenic, and osteogenic lineages was assessed. Osteogenic differentiation of hDPSCs & hPDLSCs was evaluated by Alizarin red staining. Cells assumed a polygonal shape indicating successful differentiation and also dark orange stains indicating nodules of calcification were demonstrated in both groups (left). Chondrogenic differentiation of DPSCs & PDLSCs was assessed by Alcian Blue staining (middle). Cells demonstrated morphology similar to chondrocytes rather than the typical spindle-shaped appearance of stem cells. Blue staining was demonstrated indicating the presence of sulfated glycosaminoglycans by differentiated chondrocytes. Adipogenic differentiation of hDPSCs and hPDLSCs was confirmed by Oil Red O staining (right) and demonstrated lipid droplets in differentiated cells. Accumulation of intracellular lipid droplets can be seen scattered denoting successful differentiation (scale bar 100μm)

Following in vitro differentiation, both hDPSCs and hPDLSCs were successfully differentiated into adipocytes. Intracellular lipid droplet characteristic of adipocytes was stained with Oil red O. Similarly, both hDPSCs and hPDLSCs were successfully differentiated into chondrocytes as indicated by Alcian blue staining of the deposited sulfated proteoglycans. Also, successful differentiation into osteoblasts confirmed by the presence of calcific deposits was illustrated by Alizarin red S (Fig. 4).

### Confirmation of IPC generation

#### Gene expressions of IPCs generated through differentiation of hDPSCs and hPDLSCs

**Fig. 5:**
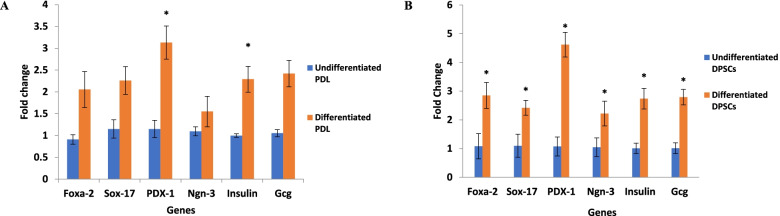
Expression of pancreatic endocrine genes in generated IPCs from hDPSCs (**A**) and hPDLSCs (**B**). Data are expressed as means ± SD. ^*^Significant change at *P* < 0.05 in comparison with the corresponding undifferentiated cells

Following the induction of both hDPSCs and hPDLSCs with the Laminin 411-enriched differentiation protocol for 14 days, the derived IPCs from hDPSCs demonstrated increased expression pancreatic endocrine genes (Foxa-2, Sox-17, PDX-1, Ngn-3, insulin, and glucagon) in comparison with undifferentiated hDPSCs as illustrated in Fig. 5a. Moreover, the generated cells from human PDLSC differentiation exhibited significant (*P* < 0.05) upregulation in the expression level of PDX-1 and insulin genes when compared with the undifferentiated hPDLSCs (Fig. 5b).

#### In vitro response to glucose stimulation

**Fig. 6:**
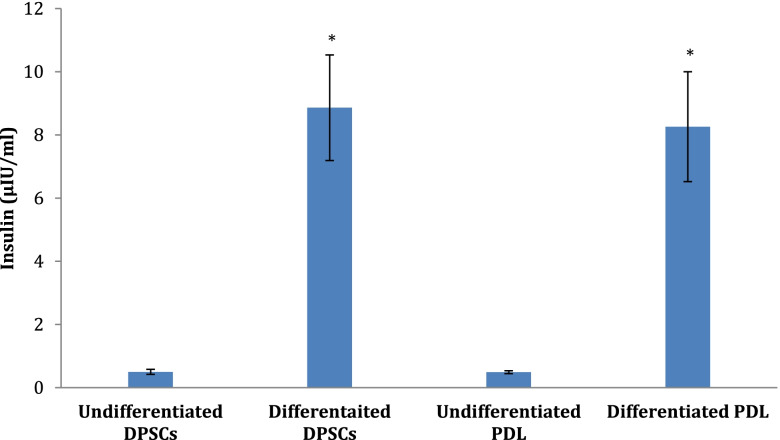
Insulin release of generated IPCs from hDPSCs and hPDLSCs. Data are expressed as means ± SD. ^*^Significant change at *P* < 0.05 in comparison with the corresponding undifferentiated cells

Data in Fig. 6 illustrated that the generated IPCs from hDPSCs and hPDLSCs secreted compelling quantities of insulin in response to escalated glucose concentration (*P* < 0.05) as to undifferentiated cells.

### Homing of transplanted IPCs into the pancreatic tissues

**Fig. 7:**
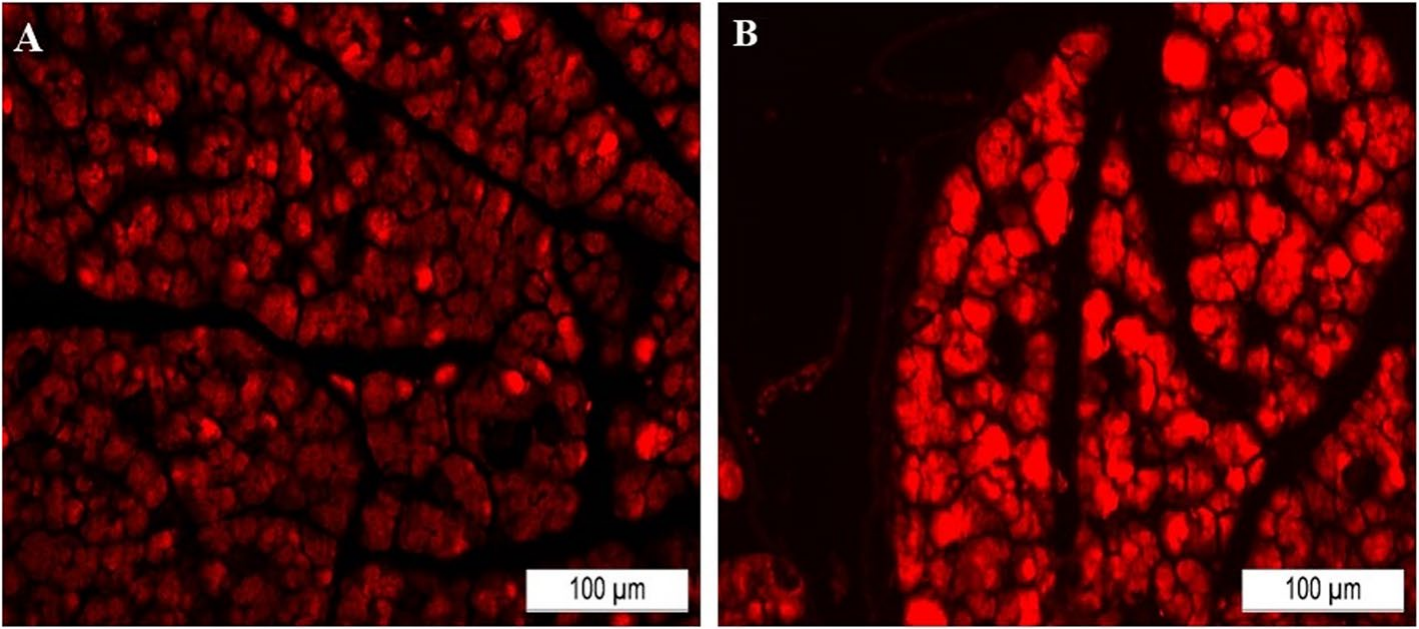
Homing of systemically administered IPCs in pancreas sections of different treated groups. **A** IPCs generated from hDPSCS. **B** IPCs generated from hPDLCSs (scale bar 100μm)

Successful homing of the injected IPCs in the pancreas of treated groups was confirmed by tracking cells labeled by PKH-26 dye. Figure 7 illustrates multiple PKH-26-labeled cells in pancreatic sections.

### Therapeutic efficacy of IPCs against DM in experimental animals

#### Influence of IPCs on diabetic indices

**Fig. 8:**
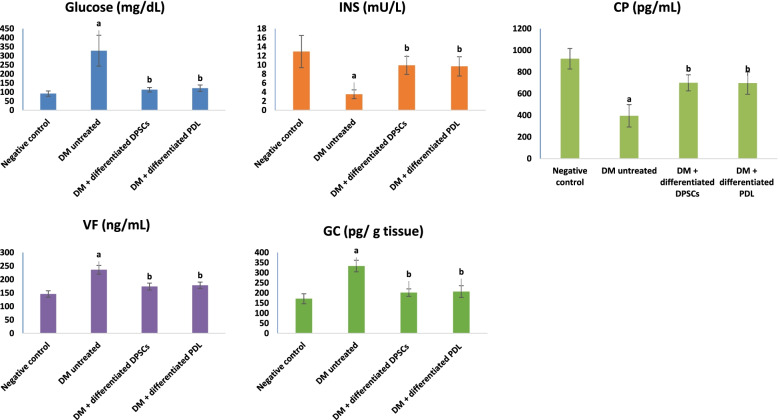
Effect of IPC implantation on (i) blood glucose, serum (ii) INS, (iii) CP, and (iv) VF as well as (v) pancreatic GC levels. Data are expressed as means ± SD of 8 rats/group. Significant change at *P* < 0.05 in comparison with the negative control group denoted by (a). Significant change at *P* < 0.05 in comparison with the untreated DM group denoted by (b)

Figure 8 represented the effect of the transplanted IPCs on the blood glucose, serum INS, CP, and VF as well as pancreatic GC levels in STZ-induced diabetes. The diabetic rats that were not treated exhibited significant (*P* > 0.05) increase in fasting blood glucose, serum VF, and pancreatic GC levels associated with significant (*P* > 0.05) decline in serum INS and CP levels as to the negative control. While transplantation of the IPCs in diabetic rats elicited significant (*P* > 0.05) decrease in the blood glucose, serum VF, and pancreatic GC levels in concomitant with significant (*P* > 0.05) increase in serum INS and CP levels versus the untreated diabetic rats.

#### Influence of IPCs on the expression level of pancreatic Foxa2, Sox17, IGF-1, and FGF10

**Fig. 9:**
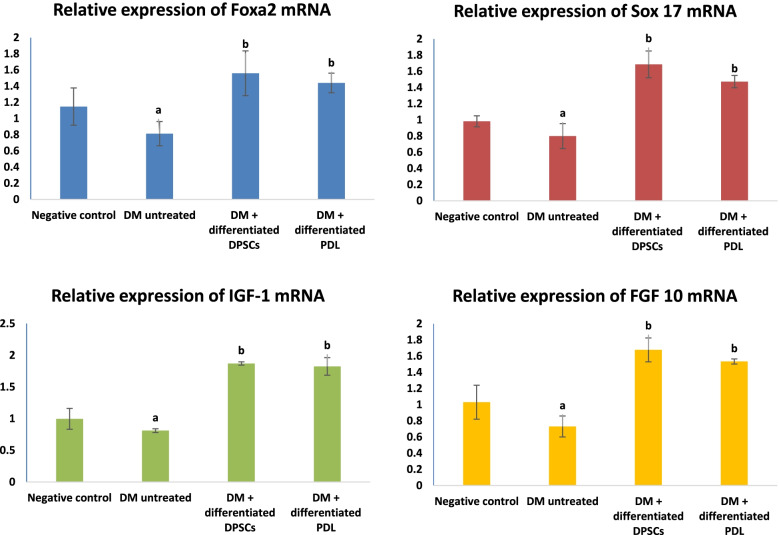
Effect of transplanted IPCs on the expression level of (i) Foxa2, (ii) Sox 17, (iii) IGF-1, and (iv) FGF 10 genes in diabetic rats. Data are expressed as means ± SD of 8 rats/group. Significant change at *P* > 0.05 denoted by (**a**) in comparison with the negative control group. Significant change at *P* > 0.05 in comparison with the untreated DM group

Figure 9 demonstrates the effect of transplanted IPCs on the expression level of pancreatic Foxa2, Sox 17, IGF-1, and FGF10 genes in diabetic rats. The diabetic rats that received no treatment demonstrated significant (*P*> 0.05) decrease in the expression level of pancreatic Foxa2, Sox 17, IGF-1, and FGF 10 genes in comparison with the control group, while transplanting IPCs in diabetic rats resulted in a significant (*P*> 0.05) increase in the expression level of pancreatic Foxa2, Sox 17, IGF-1, and FGF 10 genes when compared with the untreated DM rats.

#### Influence on histological architecture of the pancreatic tissues of diabetic rats

**Fig. 10:**
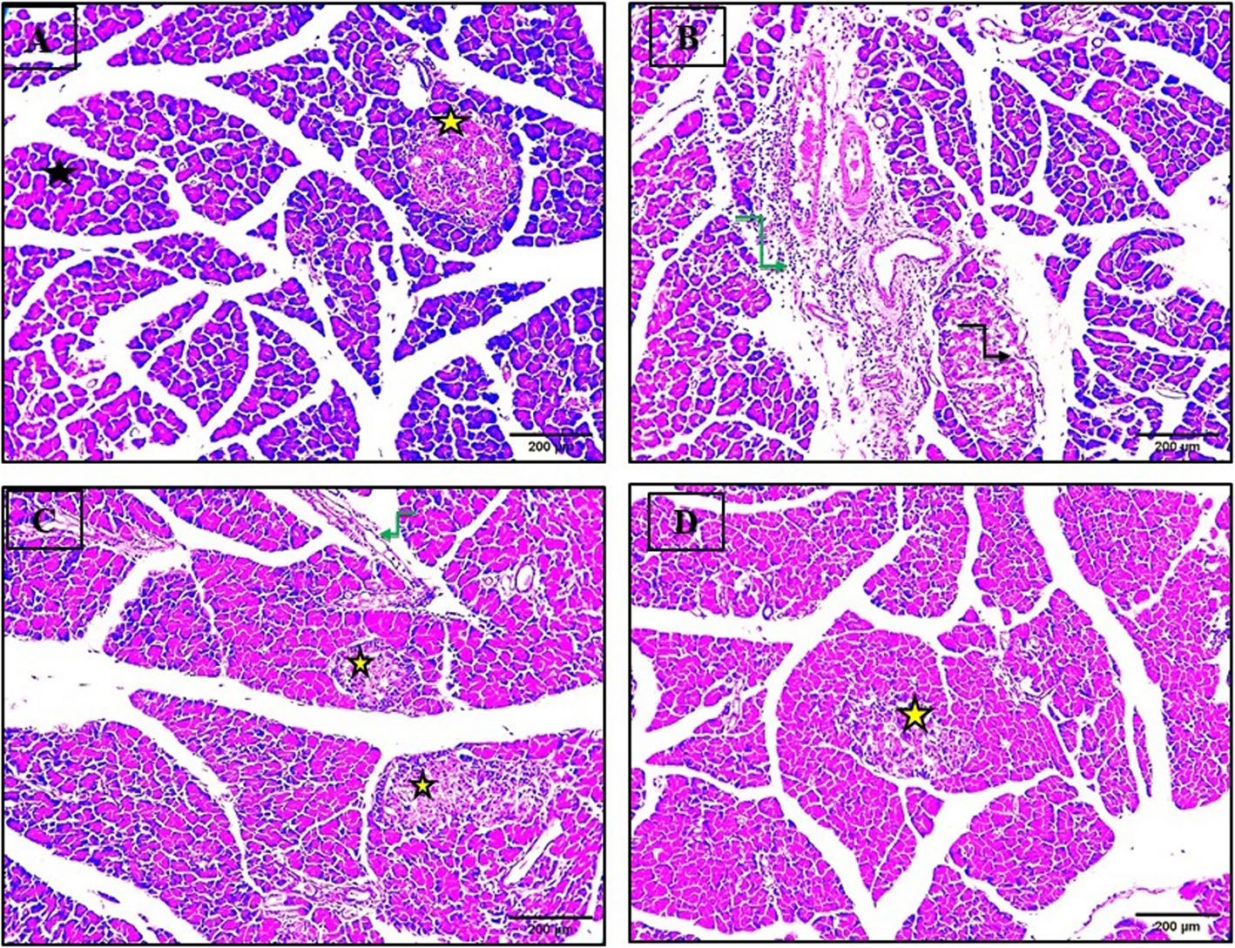
Histological section of the pancreas tissue. **A** Normal control rat showed intact pancreatic parenchyma (black star) and normal presentation of islets of Langerhans (yellow star). **B** The pancreatic tissue of untreated diabetic rat showed degenerated and disintegrated islets of Langerhans (black arrow) and heavy inflammatory cell infiltrations (green arrow). **C** The pancreatic tissue of diabetic rat infused with hDPSCs showed normal pancreatic parenchyma (yellow star) with few inflammatory cell infiltration (green arrow). **D** The pancreatic tissue of diabetic rat infused with hPDLSCs showed moderate sized islets of Langerhans (yellow star) and some inflammatory cell infiltration (scale bar 200μm)

Histological sections of the pancreas tissues of the control rats illustrated no histopathological alterations in the endocrine compartment islands of Langerhans cells which presented well-defined margins and plump islet cells. The exocrine acini together with acinar cells stained blue at their base due to the existence of a high content of RNA. Their apices stained pink where there was high content of digestive enzymes, in addition to the duct system of the exocrine portion (Fig. 10A). While the histological sections of the pancreas tissues of diabetic rats that received no treatment showed widespread destruction of Langerhans islets, the nuclei were small condensed and darkly stained, along with congested capillaries. Moreover, severe infiltration of inflammatory cells were also apparent (Fig. 10B). Histological sections of the pancreatic tissues of diabetic rats infused with IPCs generated from differentiation of hDPSCs presented normal pancreatic parenchyma with few inflammatory cell infiltration (Fig. 10C). Finally, histological examination of the pancreatic tissues of the diabetic rats treated with IPCs generated from differentiation of hPDLSCs showed moderate-sized islets of Langerhans with well-defined edges (Fig. 10D).

## Discussion

In this approach, we investigated the ability of dental-derived stem cells to differentiate into pancreatic β cells through implementing a sequential differentiation protocol originally designed for human umbilical cord mesenchymal stem cell [43]. The utilization of dental derived stem cells in this study was due to the remarkable ability of these cells to proliferate and differentiate into multiple cell types. Dental-derived stem cells are easily accessible by routine dental treatments with minimal invasive procedures. That is mainly what renders dental-derived stem cells, the cells of choice for the future of cell-based therapies. Teeth are usually extracted using local anesthesia, contrary to other sources of stem cells, which necessitates general anesthesia. Both the third molars and exfoliated deciduous teeth are considered a common source of DPSCs. Wisdom molar extraction is a frequent procedure in dental clinics. Moreover, it is the final tooth to form and is considered a valuable repository of immature cells in the former stages of odontogenesis [8, 9]. These cells are of tremendous therapeutic potential owing to their neural crest origin; additionally, they can be differentiated into various cell types, like hepatic cells and pancreatic β cells [32]. Similarly, exfoliated deciduous teeth are easily obtained from children whose age ranges from 6 to 16 years and share the same properties as those of third molars [45].

Our microscopic observation of the isolated cells indicated that MSCs derived from human dental pulp and periodontal ligament exhibited clear spindle-shaped, fibroblastic-like morphology. Such findings are supported by those of Miletić et al. [33] and Patil et al. [42] [33, 42]. In addition, both the human dental pulp and human periodontal ligament-derived MSCs are characterized by flow cytometry demonstrated positive expression of CD 73 (94.2%), CD 90 (75.8%), and CD 105 (81.9%), but were negative for CD 14 (5.56%), CD 34 (3.38%), and CD 45 (10.6%).

Pancreatic organogenesis spans a distinct interplay of factors that, together with other processes, directs stem cells to differentiate into pre-pancreatic cells and subsequently the development of a mature and functional organ. Thus, most stem cell-based differentiation strategies are mainly directed at producing mature, insulin-expressing β cells, that are reactive to glucose through mimicking the stages of pancreatic development [30, 35].

It is believed that the selection of a suitable protocol to differentiate cells is critical to the success of the differentiation [36, 60]. Additionally, the type of inductive factors used can affect the efficiency of differentiation via different pathways [3]. Differentiation can be improved through eliciting the expression of pancreatic genes such as Pdx_1_ and insulin which in turn results in increased insulin secretion in response to glucose stimulation [15, 44, 54]. We implemented an established endodermal lineage differentiation protocol that is based on the use of Laminin 411. In this protocol, laminin 411-induced differentiation of IPCs from umbilical cord-derived stem cells by way of both Pdx_1_ and Ngn_3_ pathways [43]. Results reported by this group indicated marked improvement in the differentiation ability of umbilical cord MSCs into IPCs. Also, improvement of diabetic symptoms and overall survival of diabetic rats were described.

Naturally, multiple extracellular matrix ECM proteins exist in the basement membranes of cells; however, the most eminent of which are collagen, laminins, and vitronectin. They were reported to contribute to β cell growth and viability by interacting with integrin receptors on cell surfaces. Such interactions facilitate cell migration and guide primitive streak formation and gastrulation [7]. Several research groups investigated the impact of various extracellular matrix proteins on enhancing the ability of stem cells to differentiate [13, 14, 57, 60, 61]. In fact, our group previously studied the role of recombinant human Vitronectin-N (VN) as an extracellular matrix protein on the induction of endodermal lineage differentiation from human-induced pluripotent stem cells (iPSCs). VN efficiently promoted differentiation of iPSCs into pancreatic-committed gut endoderm [5]. Similarly, Hsiao-Yun et al. reported that IPC differentiation by MSCs was remarkably improved when extracellular matrix proteins were added to the differentiation media.

In the current study, our results revealed that upon culturing dental-derived stem cells using the laminin 411-based differentiation protocol for 14 days, IPCs were successfully generated as evidenced by the upregulation in the expression of pancreatic endocrine genes (Foxa-2, Sox-17, PDX-1, Ngn-3, insulin, and glucagon). Additionally, when those cells where subjected to increasing amounts of glucose, they reacted by releasing significant quantities of insulin which further proved the successful derivation of functional pancreatic β cell.

To further assess the efficiency of the generated IPCs, we designed an experimental animal model where diabetes was induced via Streptozotocin (STZ). STZ is a diabetogenic agent, which is well known for diabetes induction in animal studies [23]. STZ induces the uptake of glucose transporter GLUT2 by pancreatic ß cells which causes further blockage of glucose-induced insulin secretion while ameliorating oxidative stress of ß cells [31]. Moreover, STZ selectively destroys β cells of the islets of Langerhans which results in the hindrance of insulin synthesis resulting in an increase in blood glucose level [18].

In this experimental setting, injection of streptozotocin in rats in the current study triggered significant elevation in blood glucose, serum VF, and pancreatic glucagon levels associated with significant reduction in serum INS and CP levels. Florence et al. [20] confirmed that administration of STZ in rats caused pancreatic swelling and degeneration of ß cells and resulted in eliciting experimental diabetes mellitus in 2 to 4 days [20]. From the molecular point of view, our data confirmed that injection of STZ caused significant downregulation in the expression levels of pancreatic Foxa2, Sox17, IGF-1, and FGF 10 genes.

At the same time, the ability of the generated IPCs to home to the impaired pancreatic tissues of diabetic rats was confirmed by PKH26 dye in concomitant with previous studies [4, 58]. Through analyzing the pancreatic tissue for positive tracing of PKH26 dye, we found that in all groups transplanted with IPCs; successful homing to the diabetic pancreas was achieved.

Our data additionally documented that the systemically administered IPCs contributed to blood glucose control in the diabetic rats through eliciting significant increase in serum INS and CP levels concomitant with significant decrease in serum VF and pancreatic GC levels. Visfatin is a unique adipo-cytokine, known as pre-B cell colony-enhancing factor or nicotinamide phosphoribosyl transferase, and plays a prominent role in insulin signal transduction, glucose homeostasis, and insulin-like effects [17, 29]. It has been suggested that elevated expression of VF is compensatory for the increased blood glucose and decreased insulin levels [37, 39, 49].

Our results also demonstrated a remarkably improved blood glucose level in diabetic rats treated with the generated IPCs. This is mainly due to the successful and constant secretion of insulin by the injected IPCs that homed in the pancreas of diabetic rats. This confirmed that the generated IPCs effectively secreted insulin which in turn succeeded in reversing the hyperglycemic state caused by diabetes. Similar research efforts have indicated that transplanting products of stem cells can reduce the symptoms of diabetes [10]. In a previous study, [62] found that insulin-secreting cells that are generated from human Warton jelly-derived mesenchymal cells successfully reversed the hyperglycemic state in diabetic rats [47]. Hsiao et al. [26] also reported that both serum CP and INS levels were significantly higher in rats after IPC transplantation [26].

Additionally, the implanted IPCs in diabetic rats resulted in an upregulated expression of pancreatic Foxa2, Sox17, IGF-1, and FGF 10. Both Foxa2 and Sox17 are considered critical regulators of definitive endoderm required for pancreas development and successful differentiation of all types of stem cells; mesenchymal, embryonic, or even induced pluripotent stem cells into insulin-producing-like cells. Jonatan et al. [29] reported the unique function of Sox17 in controlling insulin release by β cells both in normal and diabetic states [29]. As for IGF-1, several studies reported its positive influence on glucose homeostasis [21, 25, 56]. IGF-I has been reported to enhance insulin sensitivity through increasing glucose uptake peripherally and reducing hepatic glucose synthesis. It has been cited that, it is responsible for glucose metabolisms, in addition to its important role in triggering insulin sensitivity and regulating glucose uptake [2, 16, 48]. Although insulin and IGF share similar modes of action regarding glucose uptake stimulation, some studies reported that IGF is superior to insulin [41]. On the other hand, Palsgaard et al. [41] concluded that insulin resistance and hyperglycemia often witnessed in diabetic patients is due to the downregulation of IGF-1 [41]. Thus, taken together, the upregulated expression level of pancreatic Foxa2, Sox17, IGF-1, and FGF 10 reported in our study confirms the pancreatic beta cell identity of the derived IPCs.

## Conclusions

Overall, the outcomes of this research work validated that laminin 411-based differentiation protocol substantially improved the generation of functional IPCs from dental derived stem cells; nevertheless, the generated IPCs released essential levels of insulin. Furthermore, our in vivo study demonstrated that intravenous administration of the IPCs derived by this protocol effectively diminished blood glucose levels, with significant prolongation of the post-transplanted survival and recovery of the DM–relevant markers. Thus, it could be established that the generated IPCs by this differentiation protocol efficiently functioned like an undamaged pancreas both in vitro and in vivo. In conclusion, selecting the suitable protocol for IPC differentiation is crucial, together with taking into consideration the original source of stem cells since each cell type possesses distinct characteristics. Finally, IPCs generated from dental-derived stem cells in this attempt, through mimicking the in vivo microenvironment, proved to be an efficient and feasible approach in cell replacement therapy of diabetes.

### Recommendations

Although the generation of physiologically functioning beta cells from stem cells is rapidly advancing, there are yet some issues that need to be addressed in order to translate this strategy into clinical application. Future efforts need to be directed on acquiring a pure and homogenous functional population of endocrine cells free of undifferentiated cells. This could be achieved through genetically manipulating the differentiation signals or via precise gene editing. In addition, methods for scaling up the procedures while at the same time ensuring minimum variation in the final cell product need to be carefully designed. Finally, developing xeno-free strategies and tackling immune issues in addition to optimizing transplantation procedures shall eventually render this stem cell based therapy a clinical reality.

## Data Availability

The datasets used and/or analyzed during the current study are available from the corresponding author on reasonable request.

## Abbreviations

IPCs: Insulin-producing cells
MSCs: Mesenchymal stem cells
INS: Serum insulin
CP: C-Peptide
VF: Visfatin
GC: Pancreatic glucagon
Foxa2: Forkhead box protein A2
Sox17: SRY-box transcription factor 17
IGF-1: Insulin-like growth factor I
FGF 10: Fibroblast growth factor10
DM: Diabetes mellitus
ECM: Extracellular matrix
hDPSCs: Human dental pulp stem cells
hPDLSCs: Human periodontal ligament stem cells
STZ: Streptozotocin
VN: Vitronectin-N
iPSCs: Induced pluripotent stem cells
GLUT2: Glucose transporter
